# Accelerating diagnosis of Parkinson’s disease through risk prediction

**DOI:** 10.1186/s12883-021-02226-4

**Published:** 2021-05-18

**Authors:** William Yuan, Brett Beaulieu-Jones, Richard Krolewski, Nathan Palmer, Christine Veyrat-Follet, Francesca Frau, Caroline Cohen, Sylvie Bozzi, Meaghan Cogswell, Dinesh Kumar, Catherine Coulouvrat, Bruno Leroy, Tanya Z. Fischer, S. Pablo Sardi, Karen J. Chandross, Lee L. Rubin, Anne-Marie Wills, Isaac Kohane, Scott L. Lipnick

**Affiliations:** 1 Department of Biomedical Informatics, Harvard Medical School, 10 Shattuck Street, Suite 514, Boston, MA 02115 USA; 2 Department of Stem Cell and Regenerative Biology, Harvard University, Cambridge, MA 02138 USA; 3 Department of Neurology, Brigham and Women’s Hospital, Boston, MA 02115 USA; 4 Sanofi, 1 Av. Pierre Brossolette, 91380 Chilly-Mazarin, France; 5 Sanofi-Aventis Deutschland GmbH, Industriepark Höchst, 65926 Frankfurt am Main, Germany; 6 Sanofi, 50 Binney St, Cambridge, MA 02142 USA; 7 Sanofi R&D, 55 Corporate Drive, Bridgewater, NJ 08807 USA; 8 Neurological Clinical Research Institute (NCRI), Massachusetts General Hospital (MGH), Boston, MA 02114 USA; 9 Center for Assessment Technology and Continuous Health, Massachusetts General Hospital, Boston, MA 02114 USA

**Keywords:** Parkinson’s disease, Predictive medicine, Prodromal, Prediagnostic, Tremor, Gait

## Abstract

**Background:**

Characterization of prediagnostic Parkinson’s Disease (PD) and early prediction of subsequent development are critical for preventive interventions, risk stratification and understanding of disease pathology. This study aims to characterize the role of the prediagnostic period in PD and, using selected features from this period as novel interception points, construct a prediction model to accelerate the diagnosis in a real-world setting.

**Methods:**

We constructed two sets of machine learning models: a retrospective approach highlighting exposures up to 5 years prior to PD diagnosis, and an alternative model that prospectively predicted future PD diagnosis from all individuals at their first diagnosis of a gait or tremor disorder, these being features that appeared to represent the initiation of a differential diagnostic window.

**Results:**

We found many novel features captured by the retrospective models; however, the high accuracy was primarily driven from surrogate diagnoses for PD, such as gait and tremor disorders, suggesting the presence of a distinctive differential diagnostic period when the clinician already suspected PD. The model utilizing a gait/tremor diagnosis as the interception point, achieved a validation AUC of 0.874 with potential time compression to a future PD diagnosis of more than 300 days. Comparisons of predictive diagnoses between the prospective and prediagnostic cohorts suggest the presence of distinctive trajectories of PD progression based on comorbidity profiles.

**Conclusions:**

Overall, our machine learning approach allows for both guiding clinical decisions such as the initiation of neuroprotective interventions and importantly, the possibility of earlier diagnosis for clinical trials for disease modifying therapies.

**Supplementary Information:**

The online version contains supplementary material available at 10.1186/s12883-021-02226-4.

## Background

Parkinson’s Disease (PD) is the second most common neurodegenerative disorder worldwide with increasing incidence as the general population ages [1]. Existing treatments reduce disease symptoms having dramatic effects on quality of life but have not been sufficiently effective at slowing progression [2]. To effectively slow disease progression, new classes of drugs are being developed targeting genomic loci [3–7]. The primary goal of this study is to facilitate the early identification of PD onset through characterization and prediction of prediagnostic PD using widely available real-world data contained in either electronic health records or insurance claims databases.

There is evidence that clinical symptoms, including non-motor features, begin to occur several years before a PD diagnosis coinciding with the prediagnostic cellular loss [8, 9]. Early detection of these symptoms may enable earlier identification of people at high risk ultimately leading to faster diagnoses. Certain prediagnostic features, based on clinical observations, have been widely studied and include impaired olfaction, constipation, urinary disorders, disturbed sleep patterns, anxiety and depression, autonomic dysfunction, and many others [10–17]. Further insight into the first clinical presentations of these prediagnostic features, as well as others not traditionally thought to be components of prediagnostic PD, and their temporal relationships would help to delineate the pathophysiology of early PD progression. This would enable the identification of people at increased risk of developing overt PD, who could be eligible for inclusion in clinical trials of early neuroprotective strategies and ultimately preventative interventions.

Using a set of variables from the period just prior to a PD diagnosis, Schrag et al. [18] developed a logistic regression-based algorithm to effectively predict whether a person would be diagnosed with PD within 5 years. This study demonstrated the utility of real-world observational records in predicting PD diagnosis and was an important validation of statistical approaches for this phenotype. However, one limitation was their focus on the entire clinical observation window up to the day before an individual’s PD diagnosis. In this case, because the data captured included the complete medical history that was ultimately used for PD diagnosis at their subsequent visit with a neurologist, we suspected that the signal driving selectivity of this algorithm, and others built on similar methods, derived primarily from features close in time to the diagnosis itself. Therefore, we hypothesized that this algorithm was primarily influenced by patients where clinicians already suspected PD. Given that the delay to diagnosis is well-established in PD and has been shown to take a median of around 1 year [19], this would limit the impact of this diagnostic algorithm.

Furthermore, the clinical presentations used to train this algorithm are not representative of the presentations that a physician would encounter in practice. Patients were selected based on their future PD status. The resultant model was developed using presentations within 5 years of a PD diagnosis, a design consideration that biased predictions away from what would be observed in real time [20]. A clinician does not have the luxury of knowing with confidence that a particular patient is predestined to have a PD diagnosis in a specific amount of time. Given the extended prodromal period in PD, such experimental designs would artificially exaggerate the differences between diseased and non-diseased groups by selecting diseased patients at later points in their trajectory. Therefore, findings that describe how a particular risk factor is overrepresented in the “N years before PD diagnosis” cannot reliably be utilized by clinicians, and the actual effect size that would be observed of these factors cannot directly be estimated. Consequently, we sought to develop a prediction model with well-defined entry criteria to enable clinical utility based on specific clinical events. This transition from a case-control to a cohort-based model also represents an advance in the quality of evidence that the resultant predictions would represent [21].

In this study, we utilized two large health record databases to develop a model to identify which individuals progress to PD that takes into account the unique features surrounding the trajectory of PD. In contrast to existing approaches, our model ensures that predictions occur using features that are both i) significantly in advance of PD diagnoses and ii) deployable at well-defined identifiable events by clinicians in real-time. Ultimately, accurate, prospective identification of high-risk individuals would allow for earlier diagnosis, intervention, and more effective large-scale evaluation of potential therapeutics.

## Methods

### Data

The main components of this study were performed utilizing two data sources:

1. The Partners Healthcare Research Patient Data Registry, composed of electronic medical records (EMR) from approximately 6 million individuals in Massachusetts. Data used in this study covers patient records from the early 1990s through the end of 2018. Encounters from this dataset span all visits from within the Partners Healthcare system during this period.
2. A de-identified administrative claims database from a large private insurance company representing more than 75 million unique members during a period extending from January 1, 2008 through December 31, 2018. Members with zip codes in Massachusetts were excluded from our analyses so as not to overlap with the first dataset. Codes in this dataset were generated in an administrative manner based on the associated billing claim records associated with an encounter, and include all visits covered by the member’s health insurance.

In both datasets we extracted gender, year of birth, coverage or enrollment duration, zip code, ethnicity, diagnoses (in the form of International Classification of Diseases, 9th and 10th Revision codes (ICD9/10)) and procedures (in the form of Current Procedural Terminology codes (CPT)). These demographic and encounter-related codes represent the primary predictor variables for this analysis. Medication and prescriptions were not evaluated due to incomplete coverage for this population in the claims data.

We utilized a similar set of case criteria to other studies identifying PD cohorts in large medical records databases [10, 22] and further augmented them to specifically model inclusion criteria for PD clinical trials. Individuals were first required to have at least two ICD diagnosis codes for PD. The first of these codes was set as their baseline point and a second code was required within the period at least 90 days and 2 years following their baseline point. Importantly, our requirement of 2 diagnoses removes likelihood of technical errors with the temporal separation ensuring a consistent PD diagnosis upon a follow-up appointment but does not cover the imprecise diagnostic journey of a person with suspected PD. As such, it is critical to understand that our model accelerates the prediction of PD but not the prediction of who may subsequently progress to other diagnoses such as multiple systems atrophy or progressive supranuclear palsy, which would require secondary or more specific prediction models. A minimum age of 50 at baseline was set to exclude subjects that have autosomal dominant or disease strongly driven by genetics as these subjects would not be representative of typical idiopathic PD. This also ensured that the study population was not biased by subjects where “Parkinsonism” was present through a longstanding or perinatal disease separate from PD.. Subjects were required to have at least 2 years of claims data prior to their baseline diagnosis and 2 years following in order to capture the prodromal period of the disease and to track progression. The 2 years of data prior to their baseline limits the possibility of inclusion of patients with PD that were diagnosed previously [23]. Subjects with diagnoses whose treatment or progression prior to baseline could lead to secondary PD and therefore an erroneous PD diagnosis were removed such as Schizophrenia, Encephalitis, Stroke, etc. (Supplementary Table 1). Non-PD controls were matched to cases based on age, gender, and coverage.

We later resampled the databases for anyone having either a gait and/or tremor disorder diagnosis based on ICD codes (Supplementary Table 2). Cases were defined as those patients eventually diagnosed with PD and controls set to those who did not. For both cohorts, we utilized the first diagnosis of gait and/or tremor disorder as their baseline dates. All other inclusion/exclusion criteria were repurposed using this new baseline. No matching was conducted for these tasks, as the entry criteria were well defined. Subjects with a PD diagnosis at baseline were excluded as the prediction task was already accomplished by the clinician. Additional detail describing subject/control selection criteria are provided in the supplementary methods.

### Prediagnostic PD trajectory modeling/PD progression prediction

We conducted two parallel forms of modeling to examine the trajectory of prediagnostic PD: 1) a logistic regression model using an occurrence matrix of individual features; and, 2) deep learning over a patient’s observed temporal sequence of claims. A logistic regression and deep learning model were created for each evaluated time-point corresponding to different prediction window sizes: 0, 15, 30, 45, 75, 90, 180, 270, 360, 450, 640, and 720 days prior to PD diagnosis/baseline. For each time-point, a two-year long observation window preceding the specific time-point was used. As an example, for the 75-day time-point, records between 75 and 805 days prior to the baseline were utilized in the model, while records within 75 days of baseline were excluded. The features included were patient demographic data, diagnoses (when possible ICD codes were mapped to Phenome Wide Association Studies (PheWAS) [24] codes to reduce dimensionality), procedures (both CPT & ICD), and time between data points. We later repeated these tasks to model progression to PD using the two-year window prior to first gait and/or tremor disorder diagnosis.

### Static regression model

A penalized regression model was fit to predict the diagnosis of PD using a static vector constructed of the values of demographic data and counts of diagnoses, procedures and time between data points. Each patient was characterized by a frequency vector, where each element corresponded to the number of times a particular diagnosis or procedure code was observed. Demographic terms were appended to each patient vector. For each model, codes were only counted if they were present during the specified observation window. This vector was then classified by the logistic regression model based on whether it preceded a PD diagnosis. This analysis was conducted in R using the glmnet package. An independent test set was first held out. Predictive accuracy in the training set was measured via area under the receiver operator characteristics using 5-fold cross validation. Odds ratios and 95% confidence intervals were then calculated on the entire dataset. Univariate association testing was performed using age and gender-controlled logistic regression to identify features that demonstrated an association with PD onset. This association testing was first performed with all features and then again with the features present in at least 0.5% of the population. This process was repeated independently in both the insurance claims dataset as well as the Partners research database.

### Deep learning temporal sequence model

We trained a deep recurrent neural network (RNN) using gated recurrent units (GRU) to predict the onset of PD using each patient’s sequence of interactions with the healthcare system (claim or entry into medical record). Each patient was characterized by a sequence of vectors, where each vector represented a statistical profile of the diagnosis or procedure code received during an encounter. Demographic information at the time of encounter was appended to each vector. For each model, only codes present during the specified observation window were included. This sequence was then classified by the deep learning model based on whether it preceded a PD diagnosis. Sequences for the RNN were constructed using temporal embeddings trained from a separate cohort of one million individuals older than 50 [25]. Temporal sequences were constructed by interleaving tokens signifying the time between events with tokens representing the events themselves. A co-occurrence matrix was created over all tokens, where events that happened within 7 days of each other were said to co-occur. This window was chosen because events that are temporally close are likely to reflect simultaneous aspects of patient physiology. This matrix was then factored to produce a unique embedding vector for each token. Given an observation window, temporal sequences of events with the window were created for case and control individuals, using the previously created embeddings. Sequences were clipped or padded to a length of 1200 tokens to ensure equal lengths between individuals, with clipping occurring on the earliest events in a window when necessary. Sequences were classified by a deep GRU recurrent neural network in Keras using Tensorflow backend. No specific feature collection was conducted, all events that corresponded to a well defined PheWAS or CPT code were included in the model. Models were retrained from scratch using randomly selected train, and validation splits over an independently held out test set to produce confidence intervals. Neural network models were trained only in claims data due to the large amount of data required to construct embedding vectors.

### Comparison between predictive models trained using different data modalities

We compared the predictive model trained using EMR data to the model trained using administrative claims data in two ways: 1) comparing the performance of the model outputs and 2) comparing the features driving the model performance between the two different models. This comparison of relative feature importance was performed by first calculating the Pearson correlation of each data modality separately. This was compared to the correlation of feature importance between the two different data modalities.

### Comparative diagnosis prevalence

Trends in comparative diagnosis prevalence were identified by first identifying a set population of PD cases and age/gender matched control individuals with coverage prior to and after each particular window. For every given time point, defined as the 365 days relative to the point itself, and a given diagnosis, the prevalence of that diagnosis within that window was computed. Prevalence was computed for PD case and control populations separately. For example, a tremor frequency of 0.08 among cases at day 730 implies that 8.0% of PD cases had a tremor diagnosis between 730 and 365 days prior to their PD diagnosis.

## Results

### Cohort demographics

Table 1 describes the demographics of the EMR and Claims based cohorts, stratified by the PD case status. The EMR dataset contained records from 22,102 individuals, while the Claims dataset contained records from 28,216 individuals. Age of first diagnosis was slightly higher in the Claims cohort but was over 70 in both datasets. Our cohorts align with accepted estimates of PD incidence in the population [26]. Population statistics between cases and matched controls largely align between the EMR and Claims data though the latter population is slightly younger (owing to the transfer of individuals above 65 to Medicare) and has more extended terms of coverage due to the nature of the data sources. EMR records only capture an individual’s interactions with that particular hospital system, while claims records capture all of an individual’s paid interactions while they were insured.

**Table 1:** Population statistics between cases and matched controls in EMR and Claims Data. Ethnicity data was only available for a subset of patients

	EMR		Claims	
	PD Cases	Matched PD Controls	PD Cases	Matched PD Controls
Total	3251	18,851	5131	23,085
Male (%)	1903 (58.5)	11,131 (59.0)	3151 (61.4)	14,177 (61.4)
Age at First Observation (STD)	63.15 (10.78)	63.94 (10.909)	69.01 (10.33)	68.71 (10.34)
Age at Baseline (STD)	72.48 (9.33)	72.64 (10.39)	73.70 (10.23)	73.69 (10.23)
Fraction White (*data available in 20% of claims records) (Count)	87.6 (2847)	87.8 (16533)	81.8* (980)	80.6* (4237)
Percentage African American (*among available) (Count)	2.4 (78)	3.44 (647)	1.92 (23)	4.25* (223)
Percentage Hispanic (*among available) (Count)	2.4 (78)	1.47 (276)	3.42 (41)	2.85* (150)
Percentage Asian (*among available) (Count)	1.66 (54)	1.01 (207)	2.84 (34)	2.85* (150)
Percentage Other Race (*among available) (Count)	5.93 (193)	6.21 (1170)	10.01* (120)	9.41* (495)
Enrollment Months (STD)	209.08 (78.66)	186.77 (75.62)	106.51 (17.84)	104.86 (18.62)
Enrollment Months Prior to Baseline (STD)	111.88 (72.57)	104.29 (68.90)	61.49 (17.59)	64.95 (18.47)
Enrollment Months After Baseline (STD)	97.68 (58.94)	82.99 (51.43)	45.14 (16.06)	40.04 (13.44)

### Parkinson’s disease trajectory is characterized by a prodromal period

**Fig. 1:**
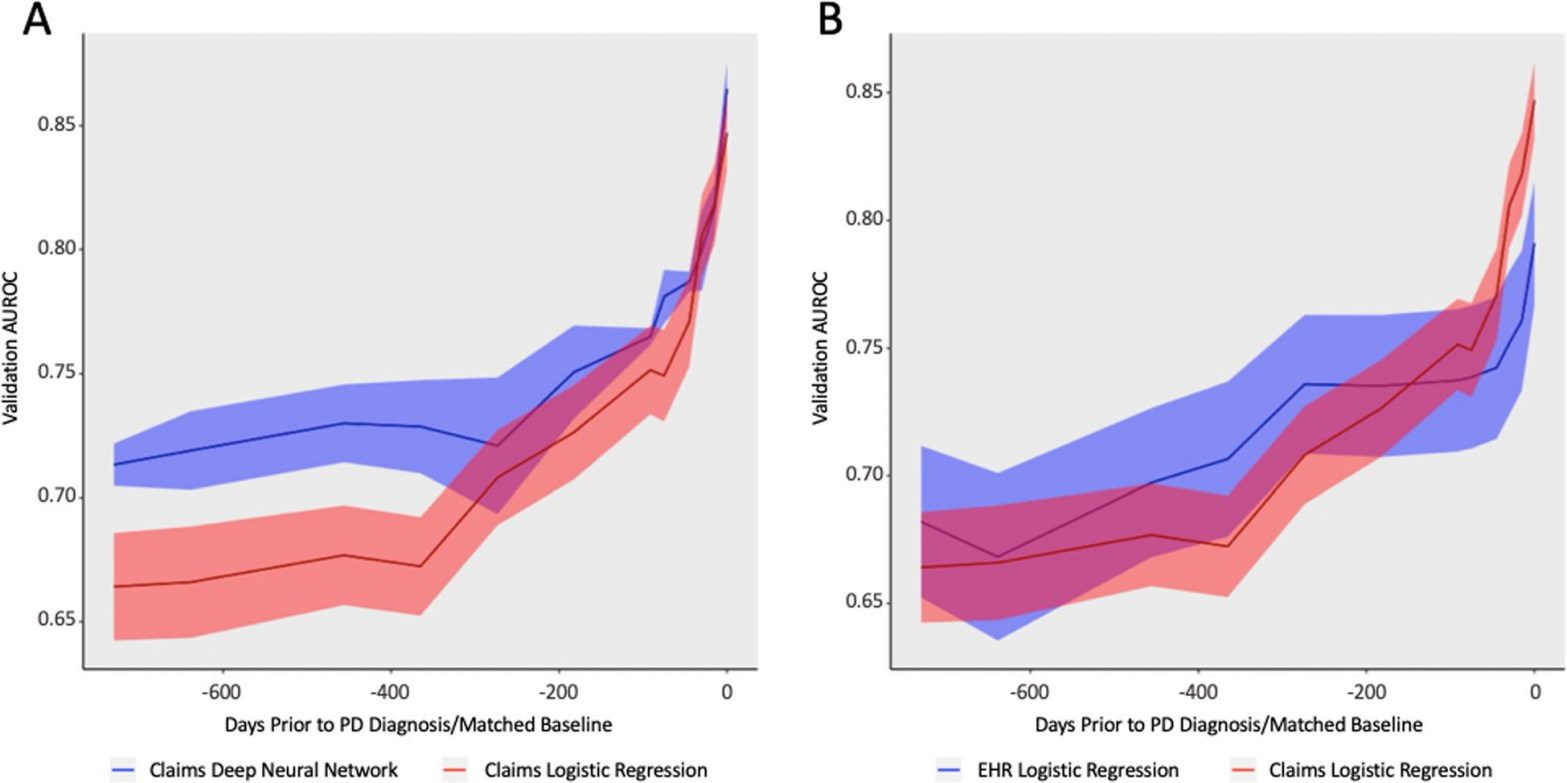
Area under the ROC Curve predicting PD onset at various points prior to PD diagnosis. **a** Logistic Regression vs. Neural Network in Claims **b** EMR vs. Claims Logistic Regression

We began by constructing two prediction algorithms, one linear and one non-linear, for future PD diagnosis utilizing 2 years of observations prior to the PD diagnosis in cases and matched controls. In contrast to prior models, we sequentially compared different time periods before the PD diagnosis date. We found a significant spike in prediction accuracy as the size of this window was reduced, which reached a maximum immediately prior to the PD diagnosis (Fig. 1a, b). We found that the accuracy of the deep neural network and a logistic regression model trained on identical claims data converged as the diagnosis date approached, implying that the most relevant signal for that time period was additive, with linear relationships between clinical events (diagnoses and procedures) driving predictions of PD status. In contrast, prediction accuracy at earlier time points appeared to be driven by non-linear, complex relationships between factors that only neural networks could resolve. The increase in performance closer to PD diagnosis date by both prediction models indicated the existence of a pre-diagnostic window during which motor symptoms were present but the diagnosis had not yet been made. Clinicians have described a time period immediately prior to diagnosis ranging between 3 months to 1 year [19] where PD is suspected and the patient is referred to neurologists or subjected to more rigorous clinical evaluation before a formal PD diagnosis is rendered. Consequently, the strong performance of classifiers that include this period may be illusory: the models draw signal from the actions of clinicians who already suspect PD. We find that the dominant features of this window include diagnoses of abnormality of gait, as well as diagnoses corresponding to tremor disorders (abnormal involuntary movements, essential tremor) (Table 2), which likely represent proxy diagnoses for PD prior to a neurologist or specialist confirming the diagnosis. Other features represent traditional, well-known, prodromal features of PD such as depression and constipation while others are less traditional such as malaise and fatigue, pain, and type 2 diabetes. To take advantage of these observations, we sought to construct models using diagnoses represented in Table 2 as new engagement points for deploying prediction models to then enable accelerated diagnosis of PD. We specifically selected gait and tremor disorders for the first set of engagement or index points for future analysis due to their comparatively extreme odds ratios. However, the remaining diagnoses, either alone or in combination, represent alternative points that could have been chosen.

**Table 2:** Top 20 diagnoses for predicting PD immediately prior to PD diagnosis

Description	OR	FDR Adjusted *P* Value	PD Prevalance	non-PD Prevalance
Abnormality of gait	8.31	2.80E-188	15.91%	2.05%
Abnormal involuntary movements	55.96	1.49E-168	19.67%	0.35%
Malaise and fatigue	3.53	2.33E-112	20.78%	6.27%
Essential tremor	38.04	1.96E-79	8.30%	0.23%
Depression	4.07	2.96E-76	10.96%	2.94%
Back pain	2.53	2.98E-62	16.11%	7.34%
Essential hypertension	1.57	3.41E-57	42.66%	31.15%
Dizziness and giddiness (Light-headedness and vertigo)	3.08	2.08E-54	12.11%	4.25%
Anxiety disorder	3.51	1.72E-51	8.51%	2.67%
Major depressive disorder	4.16	1.73E-47	4.74%	1.17%
Pain in joint	1.98	6.84E-46	21.10%	12.25%
Constipation	3.52	5.03E-42	7.73%	2.39%
Spondylosis without myelopathy	2.68	5.19E-40	11.21%	4.13%
Lack of coordination	10.97	1.63E-39	4.29%	0.37%
Pain in limb	2.26	6.99E-35	13.70%	6.76%
Extrapyramidal disease and abnormal movement disorders	51.01	8.70E-35	4.42%	0.09%
Abdominal pain	2.19	1.43E-33	12.52%	6.34%
Type 2 diabetes	1.78	4.41E-32	13.33%	8.94%
Urinary incontinence	3.46	9.38E-32	5.03%	1.69%

### Gait and tremor disorders highlight PD differential diagnostic window

**Fig. 2:**
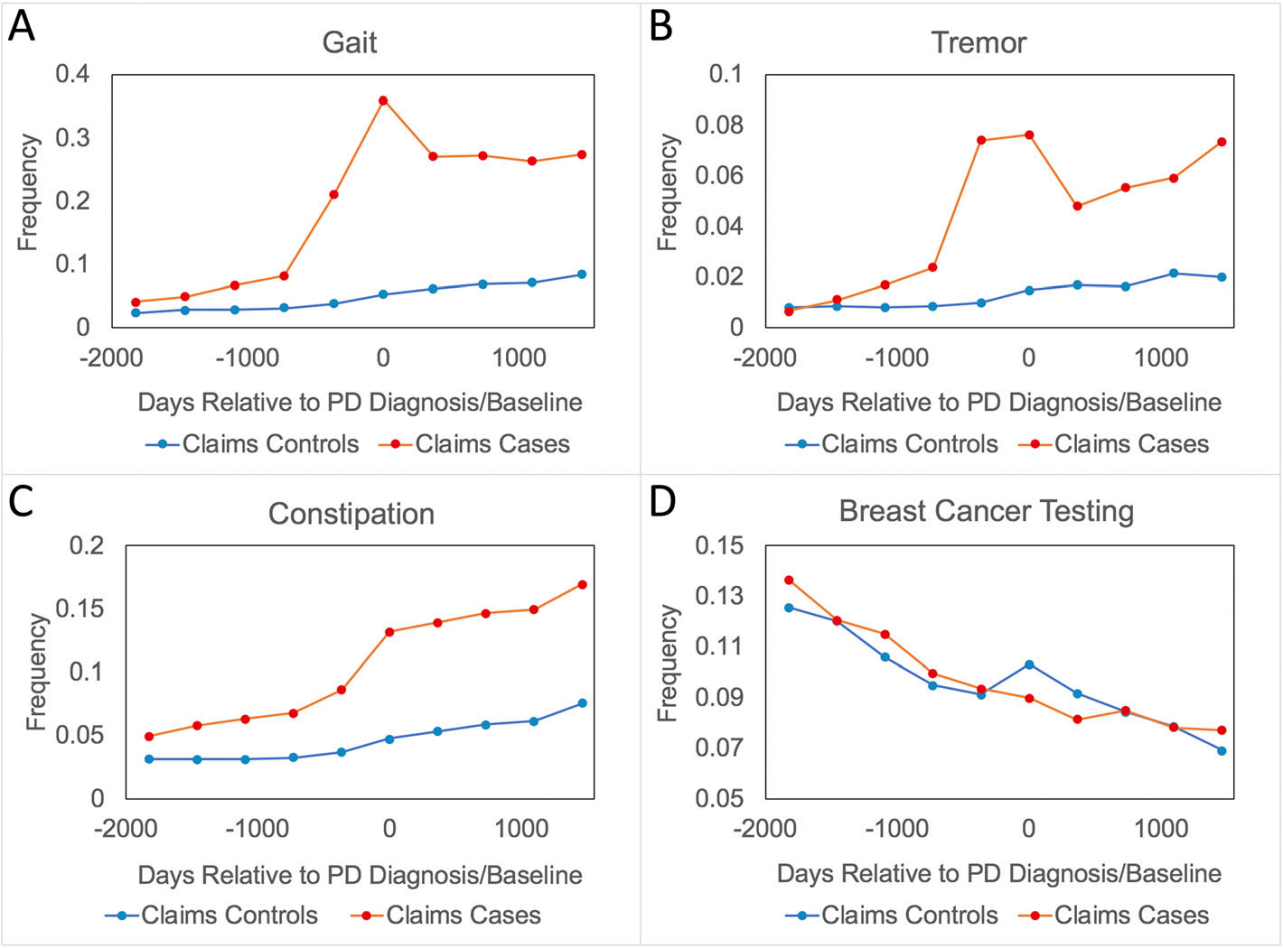
Frequency of phenotypes relative to PD diagnosis date (cases)/matched baseline date (controls). Each point represents the frequency of the phenotype among the population in the year defined at the point: a tremor frequency of 0.08 at day 730 implies that 8.0% of PD cases had a tremor diagnosis between 730 and 365 days prior to their PD diagnosis. The data in subfigures represent the population diagnosed with a (**a**) gait disorder, **b** tremor disorders, **c** constipation, or **d** breast cancer testing. Details of the ICD/CPT codes associated with each subfigure are presented in Supplementary Table 2

In order to better characterize the predictive implications and utility of this pre-diagnostic window, we examined the rates of different diagnoses relative to the PD diagnosis date corresponding to select phenotypes (Fig. 2): gait disorders, tremor disorders, constipation (a known prodromal symptom of PD), as well as a clinical event with little if any known physiological connection to PD: breast cancer screening (Supplementary Table 2). It was hypothesized that, after controlling for gender, the frequency of this clinical event among PD and non-PD patients would be roughly equivalent. Gait and tremor diagnoses were chosen based on their strength of association and the presence of sufficient patients to create PD classifiers indexed to their first diagnosis point. In the case of constipation, we found elevated rates of diagnosis prior to the PD diagnosis date, that steadily rise prior to and post PD diagnosis. A small spike at PD diagnosis is likely due to increased documentation at this critical inflection point in care. In contrast, constipation among PD controls increases more gradually over the whole window but is agnostic to the baseline date itself. This behavior is consistent with constipation’s role as a symptom of PD. Breast cancer testing, a test performed as a part of the standard of care, showed little variance between PD cases and controls throughout the entire window, consistent with the lack of evidence for a physiological association to PD. We find that gait and tremor disorders among PD cases slowly diverge from controls until a large spike approximately 1 year prior to the PD diagnosis and fall off in the years post diagnosis, likely due to their replacement with a PD code. This suggests that gait and tremor diagnoses are being used as proxy diagnoses in the runup to the PD diagnosis, consistent with the presence of a pre-diagnostic window.

### Predicting Parkinson’s disease progression from first gait/tremor diagnosis

Based on the importance of gait and tremor diagnoses in the prediagnostic models and the above finding that they are widely used as proxies for a PD diagnosis, we constructed three new cohorts where baseline classification dates were defined as i) the diagnosis of first gait or tremor disorder, ii) the first diagnosis of gait disorder only, and iii) the first diagnosis of tremor disorder only. In all three cases, all subjects were gait/tremor naive prior to their baseline. Two years of features for each subject prior to the baseline were collected. The shift from a predictor based on a case-control study to a cohort study is useful in several ways. Not only are cohort studies considered a higher level of evidence [21], but the presence of a well-defined entry date allows for deployment of a predictor in clinical workflow. We used identical model architectures/parameters (both neural network and penalized logistic regression) for gait and tremor indexed models as for prediagnostic models (Fig. 1). The primary difference was the selection of the baseline point: a point in the future for the prediagnostic models, compared to a point at present for the gait/tremor models. We find that as the models are directed to focus on more specific cohorts, accuracy declines, in both claims and EMR, as well as between both logistic regression and deep neural network-based models (Table 4). The feature importance of both models trained on both data sources showed strong correlation (Pearson correlation of 0.71) between individual feature odds ratios. Furthermore, the logistic regression model trained over EMR generalized to the external Claims population with an AUC of 0.701 (95% CI: 0.698–0.704). The strongest predictor for future PD diagnosis for all three (gait or tremor, gait only, tremor only) cohorts was bipolar disorder (Table 4A, Supplementary Tables 3–4), an association that has been highlighted by other epidemiologic studies [27]. It is important to note that many Bipolar treatments (antipsychotic medications, valproic acid) are known to cause secondary Parkinsonism, which may be a reason underlying the high observed odds ratio. However, overall, the impact of bipolar on the accuracy of the model is low given the small affected population, with 2.6% of those eventually being diagnosed with PD. Other identified features align with what has previously been documented as potential risk factors for PD including major depressive disorder [28] and voice disturbance [29]. Progression into PD from gait disorders only was uniquely defined by a history of features such as urinary tract infection and chronic laryngitis, while progression from tremor disorders only was uniquely defined by parasomnia. While both gait and tremor are known to be early symptoms of PD, the distinction that the presence of these additional diagnoses may contribute towards risk in these cohorts and may indicate differences between two subsets of disease.

We examined the strongest performing model (Table 3A), the neural network predicting PD progression from either first gait or tremor in more depth (Tables 3B and 4). For this model, we examined the average days-in-advance that the model predicted PD for individuals who truly went on to experience a PD diagnosis on record at various false positive rate (FPR) thresholds. While the mean days saved declined slightly as the FPR threshold was increased, the average was still in excess of 300 days with an FPR rate of 0.01. This indicates that model performance is not dominated by individuals who immediately go on to develop PD after a gait or tremor diagnosis, and that among this selective cohort, early diagnosis is feasible.

**Table 3:** A) Claims, EMR Prediction accuracy at first gait or tremor, first tremor, and first gait. B) Analysis of advance prediction time at various FPR thresholds for first gait or tremor Deep Neural Network Model

**A)**									
	Claims					EMR			
	Demographics			Validation AUROC (95% Confidence)		Demographics			Validation AUROC (95% Confidence)
	Cohort Size	Percent Progressing to PD	Average days to PD (STD)	Deep Neural Network	Logistic Regression	Cohort Size	Percent Progressing to PD	Average days to PD (STD)	Logistic Regression
First Gait or Tremor	8475	2.43	469 (493)	0.874 (0.869–0.879)	0.803 (0.791–0.816)	1349	3.08	548 (517)	0.804 (0.792–0.816)
First Gait Only	3925	1.37	575 (521)	0.769 (0.759–0.780)	0.791 (0.772–0.809)	694	2.23	606 (530)	0.714 (0.679–0.750)
First Tremor Only	4550	6.69	377 (447)	0.698 (0.679–0.718)	0.697 (0.674–0.719)	681	5.24	479 (490)	0.757 (0.730–0.784)
**B)**									
False Positive Rate Threshold	Observed False Negative Rate	Mean Days Accelerated Diagnosis (STD)							
0.90	0.00	377 (399)							
0.80	0.01	375 (397)							
0.70	0.03	369 (395)							
0.60	0.04	368 (396)							
0.50	0.07	360 (390)							
0.40	0.12	348 (384)							
0.30	0.18	339 (376)							
0.20	0.26	334 (372)							
0.10	0.33	322 (371)							
0.01	0.44	303 (369)

**Table 4:** A) Strongest positive features in first gait/tremor cohort B) Difference in PD progression odds ratio between deep learning prodromal cohorts (Fig. 1) and gait/tremor cohorts (Table 2)

**A)**				
Description	Gait/Tremor OR	Gait/Tremor Adjusted *P* Value	Percent of non-PD patients with history of feature	Percent of PD patients with history of feature
Bipolar disorder	3.392	1.04E-88	1.54	3.42
Major depressive disorder	1.628	1.44E-27	5.95	6.64
Voice disturbance	2.04	8.74E-21	2.10	4.15
Memory loss	1.725	5.39E-18	3.46	6.39
Other non-epithelial cancer of skin	1.334	4.14E-17	10.2	16.25
Senile cataract	1.233	1.75E-16	24.2	31.63
Other persistent mental disorders due to conditions classified elsewhere	1.944	1.33E-14	1.53	3.32
Actinic keratosis	1.219	1.06E-12	18.2	26.7
Urinary incontinence	1.414	1.16E-12	6.53	9.00
Depression	1.293	6.30E-12	13.8	14.78
Symptoms concerning nutrition, metabolism, and development	1.434	2.70E-10	4.78	7.25
Frequency of urination and polyuria	1.269	1.47E-09	10.9	15.4
Malaise and fatigue	1.133	5.64E-07	34.6	37.9
Seborrheic dermatitis	1.475	8.92E-07	2.92	4.58
Inflammation of eyelids	1.325	1.06E-06	5.86	7.90
**B)**				
Description	Prediagnostic OR	Prediagnostic Adjusted *P* value	Gait/Tremor OR	Gait/Tremor *P* Value
Mental Health Diagnoses				
Major depressive disorder	3.10	2.28E-103	1.63	1.43E-27
Mood disorders	3.62	4.54E-18	1.77	1.50E-05
Bipolar	5.68	1.82E-73	3.39	1.03E-88
Depression	2.57	3.73E-109	1.29	6.29E-12
Anemia-related Diagnoses				
Other anemias	1.13	0.412	0.74	2.67E-18
Iron deficiency anemia secondary to blood loss (chronic)	1.61	3.48E-05	0.77	0.028
Other Diagnoses				
Constipation	2.24	4.42E-72	1.17	0.001
Frequency of urination and polyuria	1.66	4.96E-39	1.26	1.47E-09
Urinary incontinence	2.11	2.56E-46	1.41	1.16E-12
Hypersomnia	2.61	3.65E-12	1.43	0.010
Hypotension NOS	1.97	3.53E-19	0.8	0.054
Dizziness and giddiness	2.29	2.35E-133	1.08	0.025

Upon review of the results, we highlighted sets of diagnoses that were significantly different between the first prediagnostic model and the gait and tremor cohort model (Table 4B). In particular, the odds ratio directionality of anemia and hypotension reversed when evaluated in the presence of first gait/tremor, meaning that these diagnoses were no longer predictive of future PD. Similarly, while constipation is a known symptom of prediagnostic PD [26], it is less useful at predicting who will progress to PD from gait/tremor than in the original cohorts. These results suggest that distinct trajectories into PD may be present, including trajectories characterized by gait or tremor disorders. Further analysis motivated by these findings, outside the scope of this article, may be warranted to evaluate differential subtypes prior to a PD diagnosis. These findings also suggest that the controls defined in gait/tremor indexed cohorts represent a distinct population from traditionally defined PD controls, and that the true real-world PD progression prediction task is sensitive to the particular comparisons that a clinician is making.

## Discussion

Early identification of PD for the purpose of disease modification remains a major unmet need. One of the potential reasons for this is the advanced disease state of the studied [30] populations. The present study bridges this gap by providing a novel approach to identify the population with gait or tremor diagnosis at risk of “converting” to PD, before marked symptoms [31]. We identify that gait and tremor associated diagnoses both i) manifest as a potential form of information leakage in existing models and ii) represent a practical opportunity for accelerating PD diagnoses. Our findings provide support to the tremor-predominant and postural instability and gait disorder (PIGD) clinical phenotypes of PD defined by the field [32]. The use of a well-defined entry point serves as a real-world event from which the model can accelerate diagnosis and are a key requirement for actual deployment of machine-learning based predictive models. Of the features selected in Table 2, gait and tremor represented the most obvious potential index points. This idea could be extended to the other features uncovered through this analysis, such as malaise and fatigue or depression. This approach where prospectively identifiable engagement or index points are selected, based on one or a combination of diagnoses, represents a paradigm shift for how predictive algorithms are designed and deployed. In contrast, we find that models that utilize all features prior to the diagnosis date in cases rely on features very close to the diagnosis date itself (as few as 15 days in advance of the true diagnosis), and so are less likely to provide clinical utility. This main original contributions of this work are: (1) the unbiased characterization of prediagnostic PD, by utilizing a well curated cohort of PD patients and matched controls; (2) the mapping of the temporal relationships of prediagnostic features to evaluate which diagnoses define the later stage of this period (i.e. the “pre-conversion” or suspected PD that we defined as a pre-diagnostic window); and (3) the deployment of novel machine learning approaches to develop a clinically deployable model for predicting which patients will progress into PD. Implementation of this strategy would facilitate earlier diagnosis and, ultimately, preventative interventions.

The presence of a pre-diagnostic period has complicated and obfuscated attempts to develop predictive models for PD using standard machine learning [17, 33, 34] approaches. Models, such as those proposed by Schrag, et al. [18], that included the pre-diagnostic window all had AUROC values between 0.8 and 0.85, despite the large differences in input data and imputation methods. Hand curated factors and simple linear models performed roughly as well as highly complex neural networks with access to a comprehensive record of interactions with the health care system. This observation implies that within this period, signal is overwhelmingly dominated by prodromal signals, and that the signal here is illusory: physicians likely already suspect PD in most of the true positive cases. In order to establish clinical utility for decision support surrounding PD, it is critical for predictive models to report at critical times in care, rather after a doctor already suspects a diagnosis. While, within populations already diagnosed with PD, gait and tremor are often seen as indicators of progression, we found that many patients with these diagnoses experienced extended periods prior to their first PD diagnosis. Even though retrospectively, one can say these individuals were clearly progressing through Braak stages, this was not evidence enough to the providers at the time to make a PD diagnosis. This modelling approach can therefore accelerate PD diagnoses by nominating high risk patients for confirmatory encounters with specialists at earlier points in time. Finally, our results demonstrate that not only are models that utilize features in the prediagnostic window are unlikely to accelerate PD diagnosis, but that predictive performance significantly declines when the prediagnostic window is removed. Models that are designed to target well-defined populations are required to have any potential for practical real-world deployment [35].

One way to address this bias is through restricting the scope of a predictive model to a more homogenous cohort defined by a specific inflection point in their health. By identifying that the pre-diagnostic period is, for many, characterized by an initial gait and tremor disorder, we avoid the biases that stem from attempting to determine if a particular model is appropriate for a particular patient. It is feasible for a physician to determine if a patient has their first gait or tremor disorder whereas it is unrealistic that a physician can predict if a patient is within 5 years from a diagnosis of PD. This ‘specificity-first’ approach can also yield insights into the heterogeneity of the disease state: as mentioned before, PD can be thought of as a syndrome with numerous subtypes. An example of a subtype can be seen by the way gait/tremor defined PD trajectory behave in a different manner than PD as a whole. The algorithm proposed by Schrag et al. [18] nominates an additive relationship between various factors, among them dizziness, hypotension, gait, and tremor. In contrast, at the time of a first gait or first tremor diagnosis, we found hypotension was no longer predictive of PD onset and dizziness had only a very weak effect. This suggests that among gait/tremor defined PD, an algorithm agnostic to latent PD subtypes may overestimate risk of progression among some patients.

Our study has several limitations driven by the use of real-world data collected primarily from billing and patient care. To mitigate this, we first used a data-driven approach to define a sufficient quiescence period prior to de novo PD diagnoses. Despite this, there is no guarantee that an individual may not have received a PD diagnosis either prior to appearing in the data (first visit or enrollment into insurance coverage) or outside of the data (in another insurance plan or health system, or through prescriptions, which were not included in this study). Unfortunately, our study was unable to include prescription data to exclude drug-induced Parkinson’s cases. The limited availability of medication data resulted in our inability to definitively remove individuals with histories of drugs that could drive secondary Parkinsonism. Finally, the pre and post-baseline record restrictions that we implemented to ensure the integrity of our cohorts would serve to bias our analysis towards populations with extended lengths of coverage.

## Conclusions

Our study has three central conclusions. First, we perform a retrospective analysis to characterize the prediagnostic period in PD by highlighting important prodromal features. These enable us to quantify the influence of this period on predictive models and show how a study design based on future events can result in artificially strong predictive performance. Second, in characterizing this period, we show that gait and tremor disorders represent anomalous patterns of diagnosis or differential diagnosis, suggesting the existence of a population of PD patients whose diagnoses can be accelerated. Third, we develop and validate a prospectively usable predictor for PD among this population and present evidence of unique comorbidities and risk factors enabling stratification of individuals affected by PD. These findings enable more nuanced predictive algorithms that better resemble the patient populations that physicians are likely to encounter in practice and potentially those likely to respond to different interventions.

Overall, this approach focusing on specific clinical decisions as experienced by physicians is well suited for not only guiding clinical decision-making regarding referrals and accelerated diagnoses, but also allows for more closely aligning machine learning predictors with the infrastructure around clinical trials. Reliable risk stratification could identify eligible patients earlier while also providing a proxy endpoint that can be tracked in a continuous manner. By providing the basis for identifying distinct subpopulations and disease progression trajectories, physiological hypotheses regarding the nature of the disease can be elucidated and more precise recommendations made to clinicians.

## Supplementary Information

**Additional file 1: Supplementary Methods. Table S1.** Included and excluded codes for initial analysis. **Table S2.** Included and excluded codes for feature tracking and gait/tremor indexed analysis. **Table S3.** Tremor-only Cohort, PD associated Diagnoses. **Table S4**: Gait-only Cohort, PD associated Diagnoses.

## Data Availability

Raw patient health data is not able to be shared because of confidentiality requirements. Partners Healthcare data, now Mass General Brigham, is available to members of the Mass General Brigham community after IRB approval using the Research Patient Data Registry (RPDR), https://rpdrssl.partners.org/. The administrative claims data is not widely available.

## Abbreviations

AUROC: Area Under the Receiver Operating Curve
CPT: Current Procedural Terminology Codes
EMR: Electronic Medical Records
FPR: False Positive Rate
GRU: Gated Recurrent Units
ICD9/10: International Classification of Diseases, 9th and 10th Revision codes
NOS: Not Otherwise Specified
OR: Odds Ratio
PD: Parkinson’s Disease
PheWAS: Phenome Wide Association Studies
PGID: Postural Instability and Gait Disorder
RNN: Recurrent Neural Network
STD: Standard Deviation

## References

[1] Pringsheim T, Jette N, Frolkis A, Steeves TDL (2014). The prevalence of Parkinson’s disease: a systematic review and meta-analysis. Mov Disord.

[2] Lang AE, Espay AJ (2018). Disease modification in Parkinson’s disease: current approaches, challenges, and future considerations. Mov Disord.

[3] Hardy J, Lewis P, Revesz T, Lees A, Paisan-Ruiz C (2009). The genetics of Parkinson’s syndromes: a critical review. Curr Opin Genet Dev.

[4] Nalls MA, Pankratz N, Lill CM, Do CB, Hernandez DG, Saad M (2014). Large-scale meta-analysis of genome-wide association data identifies six new risk loci for Parkinson’s disease. Nat Genet.

[5] Chang D, Nalls MA, Hallgrímsdóttir IB, Hunkapiller J, van der Brug M, Cai F (2017). A meta-analysis of genome-wide association studies identifies 17 new Parkinson’s disease risk loci. Nat Genet.

[6] Deng H, Wang P, Jankovic J (2018). The genetics of Parkinson disease. Ageing Res Rev.

[7] Iwaki H, Blauwendraat C, Leonard HL, Liu G, Maple-Grødem J, Corvol J-C (2019). Genetic risk of Parkinson disease and progression:: An analysis of 13 longitudinal cohorts. Neurol Genet.

[8] Berg D, Postuma RB, Adler CH, Bloem BR, Chan P, Dubois B (2015). MDS research criteria for prodromal Parkinson’s disease. Mov Disord.

[9] Mahlknecht P, Seppi K, Poewe W (2015). The Concept of Prodromal Parkinson’s Disease. J Parkinsons Dis.

[10] Schrag A, Horsfall L, Walters K, Noyce A, Petersen I (2015). Prediagnostic presentations of Parkinson’s disease in primary care: a case-control study. Lancet Neurol.

[11] Darweesh SKL, Verlinden VJA, Stricker BH, Hofman A, Koudstaal PJ, Ikram MA (2017). Trajectories of prediagnostic functioning in Parkinson’s disease. Brain..

[12] Gonera EG, van’t Hof M, Berger HJ, van Weel C, Horstink MW (1997). Symptoms and duration of the prodromal phase in Parkinson’s disease. Mov Disord.

[13] Lerche S, Seppi K, Behnke S, Liepelt-Scarfone I, Godau J, Mahlknecht P (2014). Risk factors and prodromal markers and the development of Parkinson’s disease. J Neurol.

[14] Abbott RD, Petrovitch H, White LR, Masaki KH, Tanner CM, Curb JD (2001). Frequency of bowel movements and the future risk of Parkinson’s disease. Neurology..

[15] Abbott RD, Ross GW, White LR, Tanner CM, Masaki KH, Nelson JS (2005). Excessive daytime sleepiness and subsequent development of Parkinson disease. Neurology..

[16] Ross GW, Petrovitch H, Abbott RD, Tanner CM, Popper J, Masaki K (2008). Association of olfactory dysfunction with risk for future Parkinson’s disease. Ann Neurol.

[17] Postuma RB, Aarsland D, Barone P, Burn DJ, Hawkes CH, Oertel W (2012). Identifying prodromal Parkinson’s disease: pre-motor disorders in Parkinson's disease. Mov Disord.

[18] Schrag A, Anastasiou Z, Ambler G, Noyce A, Walters K (2019). Predicting diagnosis of Parkinson’s disease: A risk algorithm based on primary care presentations. Mov Disord.

[19] Breen DP, Evans JR, Farrell K, Brayne C, Barker RA (2013). Determinants of delayed diagnosis in Parkinson’s disease. J Neurol.

[20] Yuan W, Beaulieu-Jones BK, Yu K-H, Lipnick SL, Palmer N, Loscalzo J (2021). Temporal bias in case-control design: preventing reliable predictions of the future. Nat Commun.

[21] Burns PB, Rohrich RJ, Chung KC (2011). The levels of evidence and their role in evidence-based medicine. Plast Reconstr Surg.

[22] Alonso A, Rodríguez LAG, Logroscino G, Hernán MA (2007). Gout and risk of Parkinson disease: a prospective study. Neurology..

[23] Lewis JD, Bilker WB, Weinstein RB, Strom BL (2005). The relationship between time since registration and measured incidence rates in the general practice research database. Pharmacoepidemiol Drug Saf.

[24] Denny JC, Ritchie MD, Basford MA, Pulley JM, Bastarache L, Brown-Gentry K (2010). PheWAS: demonstrating the feasibility of a phenome-wide scan to discover gene-disease associations. Bioinformatics..

[25] Beam AL, Kompa B, Schmaltz A, Fried I, Weber G, Palmer N (2020). Clinical concept embeddings learned from massive sources of multimodal medical data. Pac Symp Biocomput.

[26] Poewe W, Seppi K, Tanner CM, Halliday GM, Brundin P, Volkmann J (2017). Parkinson disease. Nat Rev Dis Primers.

[27] Faustino PR, Duarte GS, Chendo I, Castro Caldas A, Reimão S, Fernandes RM, et al. Risk of developing Parkinson disease in bipolar disorder: a systematic review and meta-analysis. JAMA Neurol. 2019. 10.1001/jamaneurol.2019.3446. 10.1001/jamaneurol.2019.3446 PMC6802493 31609378

[28] Cole SA, Woodard JL, Juncos JL, Kogos JL, Youngstrom EA, Watts RL (1996). Depression and disability in Parkinson’s disease. J Neuropsychiatr Clin Neurosci.

[29] Skodda S, Grönheit W, Mancinelli N, Schlegel U (2013). Progression of voice and speech impairment in the course of Parkinson’s disease: a longitudinal study. Parkinsons Dis.

[30] Becker G, Müller A, Braune S, Büttner T, Benecke R, Greulich W (2002). Early diagnosis of Parkinson’s disease. J Neurol.

[31] Gibb WR, Lees AJ (1988). The relevance of the Lewy body to the pathogenesis of idiopathic Parkinson’s disease. J Neurol Neurosurg Psychiatry.

[32] Stebbins GT, Goetz CG, Burn DJ, Jankovic J, Khoo TK, Tilley BC (2013). How to identify tremor dominant and postural instability/gait difficulty groups with the movement disorder society unified Parkinson’s disease rating scale: comparison with the unified Parkinson's disease rating scale. Mov Disord.

[33] Fengler S, Liepelt-Scarfone I, Brockmann K, Schäffer E, Berg D, Kalbe E (2017). Cognitive changes in prodromal Parkinson’s disease: A review. Mov Disord.

[34] Postuma RB, Berg D (2019). Prodromal Parkinson’s disease: the decade past, the decade to come. Mov Disord.

[35] Shah NH, Milstein A, Bagley PhD SC. Making machine learning models clinically useful. JAMA. 2019. 10.1001/jama.2019.10306. 10.1001/jama.2019.10306 31393527

