# Supplementary material for: Accelerating diagnosis of Parkinson’s disease through risk prediction
Source: BMC Neurol. 2021 May 18;21:201. doi: 10.1186/s12883-021-02226-4 (PMC8130278; doi:10.1186/s12883-021-02226-4)
Supplement: Supplementary file 1 — Additional file 1: Supplementary Methods. Table S1. Included and excluded codes for initial analysis. Table S2. Included and excluded codes for feature tracking and gait/tremor indexed analysis. Table S3. Tremor-only Cohort, PD associated Diagnoses. Table S4: Gait-only Cohort, PD associated Diagnoses. [file 12883_2021_2226_MOESM1_ESM.docx]

**Supplemental Information:**

**Supplementary Methods:**

Subject/Control Selection Criteria

Subjects who presented with diagnoses for encephalitis, Alzheimer’s disease or similar cognitive disorders that could phenocopy a true idiopathic PD diagnosis during the window prior to baseline were removed. Subjects with any presentation prior to baseline date of schizophrenia, other Parkinson’s like disorders including metabolic neurogenic disorders (e.g. Wilson’s Disease), or other degenerative diseases that produce a clinical syndrome of parkinsonism (Multiple system atrophy, Progressive supranuclear palsy) were removed. All codes utilized are listed in Supplementary Table 1.

A control cohort was structured in a similar manner. The starting point for the Partners cohort, based on available research infrastructure Partners Healthcare Research Patient Data Registry, was a cohort of 120,000 individuals with no PD diagnoses who roughly matched the age and gender profile of the total population of individuals with at least 1 PD diagnostic code. For the Claims data, similar age and confounding diagnosis criteria were utilized, but patients were required to have no indications of PD in the 2 years following the baseline. Next, an artificial baseline point in time was established such that the distribution of available records following their baseline point matched the distribution of the same time window for the PD cohort. This was done to ensure a comparable follow-up window in which all controls must have representative data. We finally required that all subjects have at least 2 years prior to and post their baseline date, the latter being a criteria already established from the baseline point matching, and be at least 50 years of age at their baseline point. Finally, we selected a matched subset of controls to PD cases using age (within 5 years) and gender.

**Supplementary Table 1:** Included and excluded codes for initial analysis

| Initial Inclusion Criteria | | | |
| --- | --- | --- | --- |
| Disease | ICD9 | ICD10 | CPT |
| Parkinson's Disease | 332, 332.0 | G20 | NA |
| Pre-PD exclusions | | | |
| AD/Cognitive Issues | 331* | G30* | NA |
| Dementia | 290* | F03.90 | NA |
| Multiple Systems Atrophy/Progressive Supranuclear Palsy | 333.0 | G90.3, G23.1 | NA |
| Schizophrenia | 295* | F20* | NA |
| Lewy Body Dementia | 331.82 | G31.83 | NA |
| Encephalitis | 323* | G04* | NA |
| Wilson's Disease | 275.1 | E83.01 | NA |

**Supplementary Table 2:** Included and excluded codes for feature tracking and gait/tremor indexed analysis

| Feature | ICD9 | ICD10 | CPT |
| --- | --- | --- | --- |
| Screening mammography, bilateral (2-view study of each breast), including computer-aided detection (cad) when performed | NA | NA | G0202 |
| Tremor/Abnormal Movements | 781.0, 781.7, 333.1, 333.90, 333.99 | R25.0, R25.1, R25.2, R25.3, R25.8, R25.9, R29.0, G25.0, G25.1, G25.2 | NA |
| Gait Disorders | 781.2 | R26.0, R26.1, R26.81, R26.89, R26.9 | NA |
| Constipation | 564.00, 564.01, 564.02, 564.09 | K58.1, K59.00, K59.01, K59.02, K59.03, K59.04, K59.09 | NA |

**Supplementary Table 3:** Tremor-only Cohort, PD associated Diagnoses

| Description | OR | Adjusted P Value |
| --- | --- | --- |
| Bipolar | 2.03 | 8.69E-16 |
| Difficulty in walking | 1.43 | 0.000320 |
| Senile cataract | 1.16 | 0.00124 |
| Lack of coordination | 1.54 | 0.00568 |
| Voice disturbance | 1.47 | 0.00811 |
| Memory loss | 1.38 | 0.00860 |
| Other non-epithelial cancer of skin | 1.19 | 0.00860 |
| Osteoporosis NOS | 1.21 | 0.0193 |
| Parasomnia | 1.92 | 0.0348 |
| Symptoms concerning nutrition, metabolism, and development | 1.26 | 0.0380 |

**Supplementary Table 4:** Gait-only Cohort, PD associated Diagnoses

| Description | OR | Adjusted P Value |
| --- | --- | --- |
| Bipolar | 4.32 | 2.40E-49 |
| Major depressive disorder | 2.20 | 1.09E-35 |
| Other persistent mental disorders due to conditions classified elsewhere | 2.61 | 6.23E-21 |
| Urinary incontinence | 1.68 | 1.73E-16 |
| Depression | 1.55 | 2.34E-16 |
| Other non-epithelial cancer of skin | 1.40 | 2.47E-12 |
| Memory loss | 1.87 | 3.99E-12 |
| Voice disturbance | 2.21 | 4.88E-12 |
| Malaise and fatigue | 1.26 | 5.44E-12 |
| Degeneration of intervertebral disc | 1.28 | 3.79E-11 |
| Frequency of urination and polyuria | 1.41 | 3.35E-10 |
| Actinic keratosis | 1.26 | 6.65E-09 |
| Senile cataract | 1.24 | 8.73E-09 |
| Dizziness and giddiness (Light-headedness and vertigo) | 1.28 | 1.76E-08 |
| Orthostatic hypotension | 1.85 | 2.35E-08 |
| Psychosis | 1.99 | 3.80E-08 |
| Symptoms concerning nutrition, metabolism, and development | 1.55 | 4.83E-08 |
| Generalized anxiety disorder | 1.75 | 1.43E-07 |
| Syncope and collapse | 1.33 | 5.95E-07 |
| Mood disorders | 2.53 | 9.28E-07 |
| Seborrheic dermatitis | 1.68 | 3.93E-06 |
| Functional disorders of bladder | 1.57 | 3.93E-06 |
| Retention of urine | 1.36 | 5.25E-06 |
| Urinary tract infection | 1.22 | 6.61E-06 |
| Chronic laryngitis | 2.32 | 5.46e-04 |
